# Quantitative risk factor analysis of prior disease condition and socioeconomic status with the multiple myeloma development: nationwide cohort study

**DOI:** 10.1038/s41598-024-52720-1

**Published:** 2024-02-28

**Authors:** Suein Choi, Eunjin Kim, Jinhee Jung, Sung-Soo Park, Chang-Ki Min, Seunghoon Han

**Affiliations:** 1 Department of Pharmacology, College of Medicine, The Catholic University of Korea, 222 Banpodaero, Seochogu, Seoul, Republic of Korea; 2 Pharmacometrics Institute for Practical Education and Training (PIPET), College of Medicine, The Catholic University of Korea, Seoul, Republic of Korea; 3 Department of Hematology, Seoul St. Mary’s Hematology Hospital, College of Medicine, The Catholic University of Korea, Seoul, Republic of Korea; 4 Catholic Research Network for Multiple Myeloma, Seoul, Republic of Korea

**Keywords:** Cancer, Cancer epidemiology, Cancer screening, Haematological cancer

## Abstract

Early diagnosis and following management are important determinants of the prognosis of multiple myeloma (MM). However, screening for MM is not routinely performed because it is rare disease. In this study, we evaluated the association of prior disease condition and socioeconomic status (SES) with MM diagnosis and developed a simple predictive model that can identify patients at high risk of developing MM who may need screening using nationwide database from South Korea. According to multivariate logistic regression analysis, eight prior disease conditions and SES before diagnosis were shown to be predictors of MM development and selected for score development. Total prediction scores were categorized into four groups: patients without any risk (≤ 0) intermediate-1 (0.5–9), intermediate-2 (9–14), and high risk (> 14). The odds ratios for developing MM in the intermediate-1, intermediate-2, and high-risk groups were 1.29, 3.07, and 4.62, respectively. The association of prior disease conditions and SES with MM diagnosis were demonstrated and the simple scoring system to predict the MM risk was developed. This scoring system is also provided by web-based application and could be a useful tool to support clinicians in identifying potential candidates for MM screening.

## Introduction

Multiple myeloma (MM) is a plasma cell disorder and malignancy that accounts for 1% of all neoplasms and 10% of all hematologic malignancies^1,2^. Globally, approximately 170,000 new cases are diagnosed annually, and the cumulative risk of developing multiple myeloma by age 75 is estimated to be 0.25% for males and 0.17% for females^3^. Despite dramatic advances in treatment of MM and a significant increase in life expectancy, every available treatment option has inevitably resulted in relapse of disease and/or development of a refractory status^4^. Thus, MM is regarded as an incurable disease and the real-world 5-year overall survival (OS) rate of newly diagnosed MM was estimated to be 50–60%^5,6^.

MM patients present with the highest symptomatic burden at diagnosis and generally experience the poorest quality of life among all malignancies^7,8^. Accordingly, it has been generally recommended that prompt management be initiated prior to onset of severe myeloma-related symptoms^9^. The International Myeloma Working Group has identified three biomarkers as indications for anti-MM treatment, in patients with no significant MM-related symptoms: bone marrow clonal plasma cells ≥ 60%, serum involved-to-uninvolved free-light-chain ratio ≥ 100, and more than one focal lesion on magnetic resonance imaging^10^. Nevertheless, screening for MM is not routinely performed, mainly because it is rare disease. Therefore, it would be ideal if high risk group could be identified, and screening of MM could be performed on them.

Although the exact pathophysiology of MM remains poorly elucidated, clonal transformation of physiological plasma cells has been shown to depend on certain inflammation-linked cytokines^11,12^. Gaucher’s disease, for example, is an inherited lysosomal storage disorder in which lipids accumulate due to a glucocerebrosidase deficiency; the condition is characterized by a high risk of developing cancer, including MM^13^. In addition, inflammation and chronic stimulation of the immune system by lipids are closely associated with MM in patients with Gaucher’s disease^14^. Like the association between Gaucher’s disease and systemic inflammation, it is generally accepted that any prior disease condition could induce a systemic inflammatory state with elevated cytokine expression that induces hyperfunctioning plasma cells^15^.

Also, it is well known that deprived, low socioeconomic status (SES) population have a higher prevalence of multiple myeloma mostly due to occupational hazard with farmers and industrial workers, especially who had prolonged exposure to pesticides or other industrial chemicals^16,17^. Thus, we hypothesized the feasibility of a prior disease conditions- and SES-based model to predict the development of MM.

The current research aimed to first explore epidemiological differences including sociodemographic characteristics and prior disease conditions between patients with MM and the matched general population who had not been diagnosed with MM. Further, we aimed to develop a score model which predicts the risk of MM development based on prior disease conditions and SES to identify high-risk patients and provide the screening for early detection.

## Results

### Dataset

**Figure 1:**
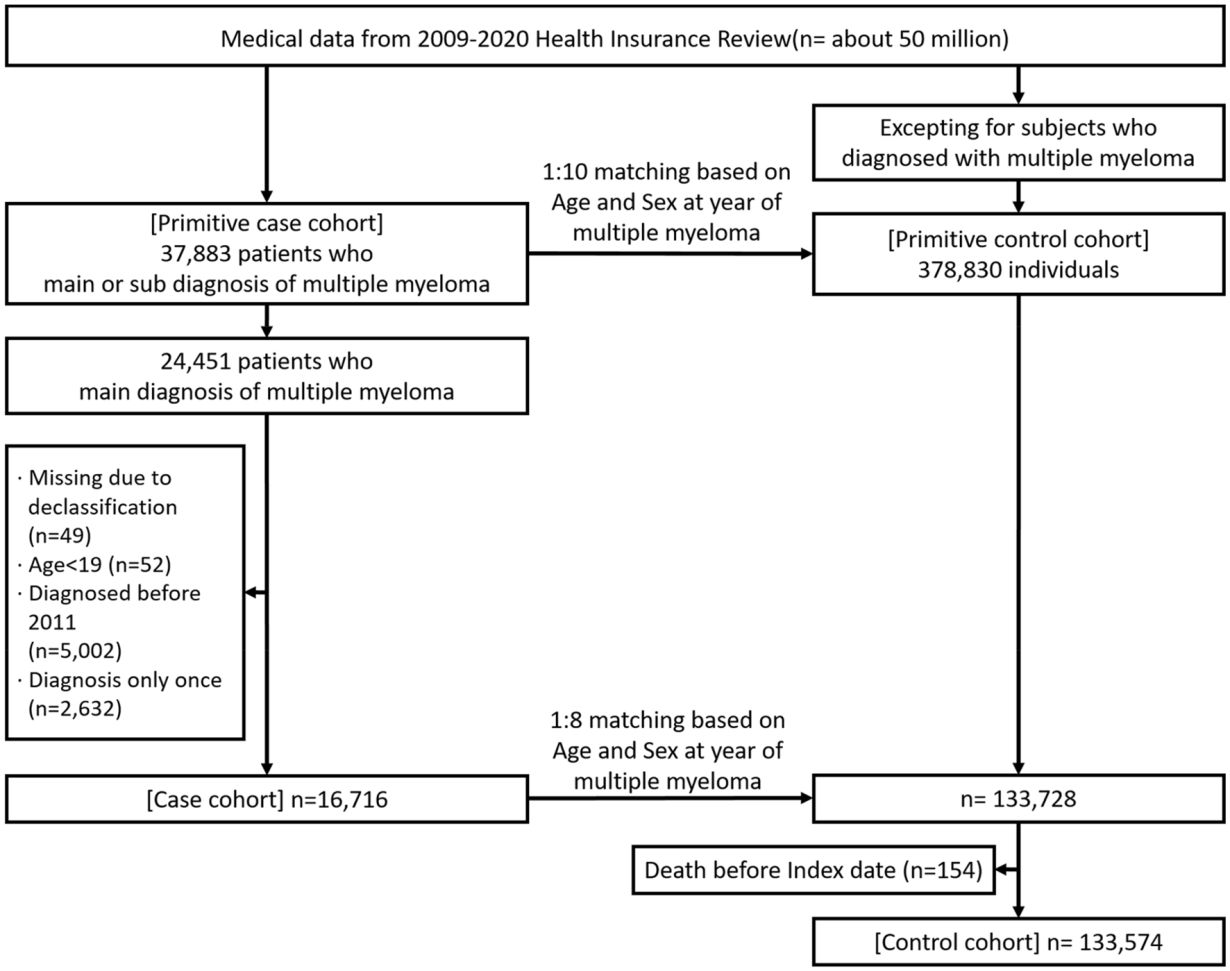
Flow diagram outlining selection of the case and control cohorts.

A total of 50 million subjects was enrolled in the Korea National Health Insurance Service (KNHIS) database and all claimed medical data between 2009 and 2020 was collected. Between 2009 and 2020, 37,883 patients were diagnosed with multiple myeloma. Among these, 17,879 patients were considered newly diagnosed patients with secure period of prior disease data collection according to the predefined protocol (i.e., main diagnosis appearing more than one time during 2011 to 2020 without any main diagnosis before 2011). The primitive control cohort included a total of 378,830 subjects, of which 133,728 were selected as final control subjects after propensity-score matching based on birth date and sex and exclusion of subjects who died before the matched index date. The study design is summarized in Fig. 1.

### Patient characteristics

The baseline demographics of both cohorts are presented in Table 1. In total, 16,716 MM patients and 133,728 individuals from the general population, defined as the case and control cohorts, respectively, were included in the analyses. The age distributions and sex ratios were similar, indicating the cohorts were well matched based on propensity scores. Among the prior disease conditions, the prevalence of congestive heart failure, autoimmune disease, chronic pulmonary disease, peptic ulcer disease, hepatic disease, renal disease, diabetes, any malignancy, and metastatic solid cancer before index date was greater in the case cohort. Also, SES was significantly different between the cohorts. A larger percentage of individuals in the case cohort had a medical beneficiary before diagnosis, and fewer percentage of patients had high SES (*p-value* < 0.001).

**Table 1:** Comparison of baseline demographics: Case cohort vs. control cohort.

Variables	Cohort		*p*-value
	Case (N = 16,716)	Control (N = 133,574)	
Age (years)*	67.1 (11.4)	67.1 (11.4)	0.870
Sex			0.972
Male	9084 (54.3)	72,569 (54.3)	
Female	7632 (45.7)	61,005 (45.7)	
Socioeconomic status**			< 0.001
Medical beneficiary	807 (4.8)	3343 (2.5)	
Low-middle	8969 (53.7)	69,336 (51.9)	
High	6639 (39.7)	57,022 (42.7)	
Unknown	301 (1.8)	3873 (2.9)	
Prior disease condition***			
Myocardial infarction	269 (1.6)	1962 (1.5)	0.157
Congestive heart failure	1282 (7.7)	7938 (5.9)	< 0.001
Peripheral vascular disease	3439 (20.6)	25,754 (19.3)	< 0.001
Cerebrovascular disease	2548 (15.2)	19,507 (14.6)	0.028
Dementia	812 (4.9)	6810 (5.1)	0.181
Hemiplegia or paraplegia	180 (1.1)	1578 (1.2)	0.236
Autoimmune disease	1124 (6.7)	7147 (5.4)	< 0.001
Chronic pulmonary disease	7512 (44.9)	53,389 (40.0)	< 0.001
Peptic ulcer disease	6796 (40.7)	50,570 (37.9)	< 0.001
Hepatic disease	3986 (23.8)	28,571 (21.4)	< 0.001
Renal disease	622 (3.7)	2245 (1.7)	< 0.001
Diabetes without chronic complication	4024 (24.1)	30,105 (22.5)	< 0.001
Diabetes with chronic complication	1896 (11.3)	13,296 (10.0)	< 0.001
Any malignancy	1604 (9.6)	10,504 (7.9)	< 0.001
Metastatic solid tumor	145 (0.9)	831 (0.6)	< 0.001
AIDS/HIV	3 (0.0)	21 (0.0)	0.745

All values are presented as number (%).

*Presented as mean (standard deviation).

**Social economic status: medical beneficiary 0; low-middle 1–18; high 19–20.

***Prior disease conditions were categorized based on Charlson comorbidity index.

Supplementary Table S1 presents data regarding the interval time to multiple myeloma diagnosis from diagnosis of prior disease condition which showed significantly higher prevalence in the case cohort. For most prior disease condition, the median interval time was around 60–70 months, whereas renal disease showed the shortest interval time, with medians of 40.

### Univariate and multivariate analyses

Eighteen variables including history of prior disease condition diagnosis and SES before diagnosis were considered relevant to the diagnosis of MM and were tested for significance on development of MM using univariate logistic regression. From this analysis, 12 prior disease conditions and SES were identified as candidate predictors (Supplementary Table S2). In the evaluation of multicollinearity among the variables considered for our multivariate logistic regression analysis, VIF did not exceed the threshold value of 5 for any predictor, indicating no significant multicollinearity concerns in our model (Supplementary Table S3). Multivariate logistic regression was then performed using those predictors, and 8 prior disease conditions and SES were selected for the final model. Among 12 prior disease conditions that were significant variables in univariate analyses, four prior disease conditions (cerebrovascular disease, diabetes without chronic complications, diabetes with chronic complications, and peripheral vascular disease) were excluded during the stepwise multivariate analysis because including them did not improve the prediction performance of the model based on AIC values. The result of multivariate analysis is shown in Table 2 including odds ratio and beta coefficient (95% CI) of each covariate. In the final model, the history of renal diseases and SES at MM diagnosis had the highest odds ratio of 2.06 and 1.76 respectively.

**Table 2:** Results of multivariate logistic regression and point allocation for predictors of multiple myeloma based on regression coefficients.

Variables	Odds ratio (95% CI)	Regression coefficient (95% CI)	*p*-value	Score assigned*
Congestive heart failure			< 0.001	
No				0
Yes	1.16 [1.09, 1.24]	0.148 [0.09, 0.22]		1.5
Autoimmune disease			< 0.001	
No				0
Yes	1.18 [1.11, 1.26]	0.166 [0.1, 0.23]		1.5
Chronic pulmonary disease			< 0.001	
No				0
Yes	1.15 [1.11, 1.19]	0.14 [0.1, 0.17]		1.5
Peptic ulcer disease			0.118	
No				0
Yes	1.03 [0.99, 1.06]	0.03 [− 0.01, 0.06]		0.5
Hepatic disease			0.001	
No				0
Yes	1.07 [1.03, 1.11]	0.068 [0.03, 0.10]		1
Renal disease			< 0.001	
No				0
Yes	2.06 [1.88, 2.26]	0.723 [0.63, 0.82]		7
Any malignancy			< 0.001	
No				0
Yes	1.16 [1.09, 1.23]	0.148 [0.09, 0.21]		1.5
Metastatic solid tumor			0.095	
No				0
Yes	1.17 [0.97, 1.41]	0.157 [− 0.03, 0.34]		1.5
Socioeconomic status			< 0.001	
Medical beneficiary	1.76 [1.62, 1.90]	0.565 [0.48, 0.64]		5.5
Low-middle				0
High	0.88 [0.85, 0.91]	− 0.128 [− 0.16, − 0.09]		− 1.5

*Based on regression coefficients.

### Score development and categorization

Score point was allocated by setting 0.5 as a unit based on the beta coefficient of multivariate logistic regression and is presented in Table 2. The allocated prediction score for each variable ranged between -1.5 and 7, with a high score indicating high risk of developing MM. Total scores were calculated as follows:

$$ \begin{aligned} Total\;score & = 1.5\left( {Congestive\;heart\;failure} \right) + 1.5\left( {Autoimmune\;disease} \right) \\ & \quad \left. { + 1.5\left( {Chronic} \right.pulmonary \, disease} \right) + 0.5\left( {Peptic\;ulcer\;disease} \right) \\ & \quad + 1 \, \left( {Hepatic\;disease} \right) + 7\left( {Renal\;failure} \right) + 1.5\left( {Any\;malignancy} \right) \\ & \quad + 1.5\left( {Metastatic\;solid\;tumor} \right) + 5.5\left( {Medical\;beneficiary} \right) - 1.5\left( {High\;SES} \right) \\ \end{aligned} $$

**Figure 2:**
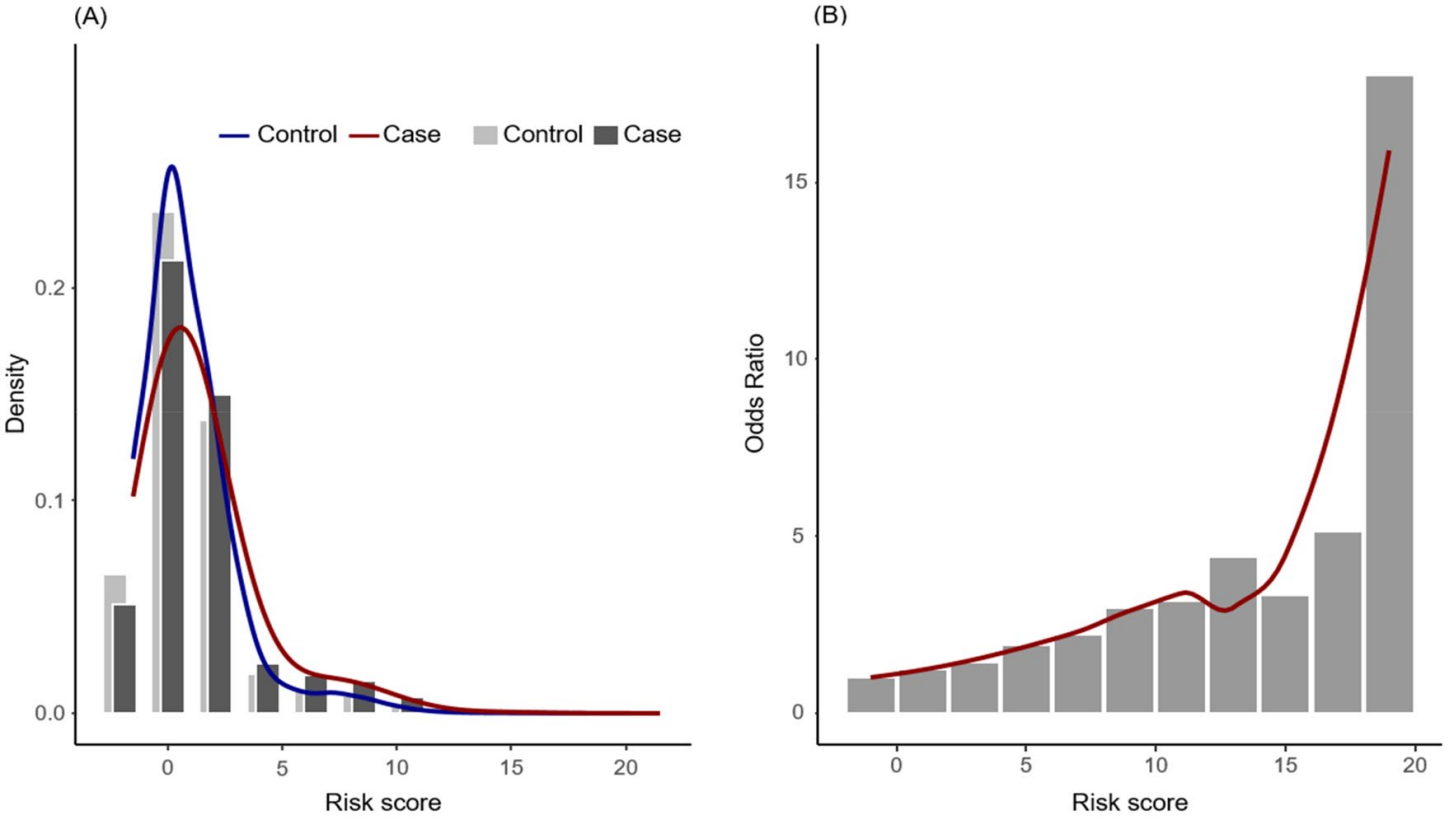
(**a**) Histogram of risk scores in the case cohort (dark grey) and control cohort (light grey*)*. The frequency distribution of risk scores in the case cohort (red line) and control cohort (blue line). (**b**) The odds ratios for multiple myeloma development according to risk score. The red line represents the loess locally smoothed relationship between the odds ratio and risk score.

The total scores of individuals in the case cohort were significantly greater than those in the control cohort (*p-value* < 0.001), and distributions are shown in Fig. 2a. The total scores of individuals ranged from -1.5 to 21.5. Figure 2b also shows that the risk of developing MM increased as the prediction score increased. Prediction scores were categorized into four groups considering the distribution and clinical efficiency: patients without risk factors (≤ 0) intermediate-1 (0.5–9), intermediate-2 (9–14), and high risk (> 14). The odds ratios for developing MM in the intermediate-1, intermediate-2, and high-risk groups compared with the group without risk factors were 1.29, 3.07, and 4.62, respectively.

### Subcohort analysis for internal validation

To address the differences in prior disease condition collection period according to index date and to validate the scoring model, sub-cohort analyses were performed for individuals who had a sufficient prior disease condition collection period more than 5 years (index date from 2014 to 2020). The prediction scores were calculated for the individuals in the sub-cohort, and the individuals were categorized according to previously defined score groups. The odds ratio of each sub-cohort was comparable to that of the original cohort, showing that the scoring model was internally valid and a collection period of one year was sufficient (Table 3).

**Table 3:** Risk evaluation in categorized groups for the original cohort and sub-cohort.

	Score	Cohort (2011–2020)		Sub-cohort (2014–2020)	
		OR*	*p*-value	OR*	*p*-value
Intermediate-1	0.5–6	1.29 [1.25, 1.34]	< 0.001	1.28 [1.23, 1.33]	< 0.001
Intermediate-2	6–14	3.07 [2.67–3.52]	< 0.001	2.88 [2.48, 3.32]	< 0.001
High	> 14	4.62 [3.14, 6.68]	< 0.001	4.16 [2.77, 6.11]	< 0.001

*Based on the group without any risk factors (Score ≤ 0).

### Web-based application for score model

**Figure 3:**
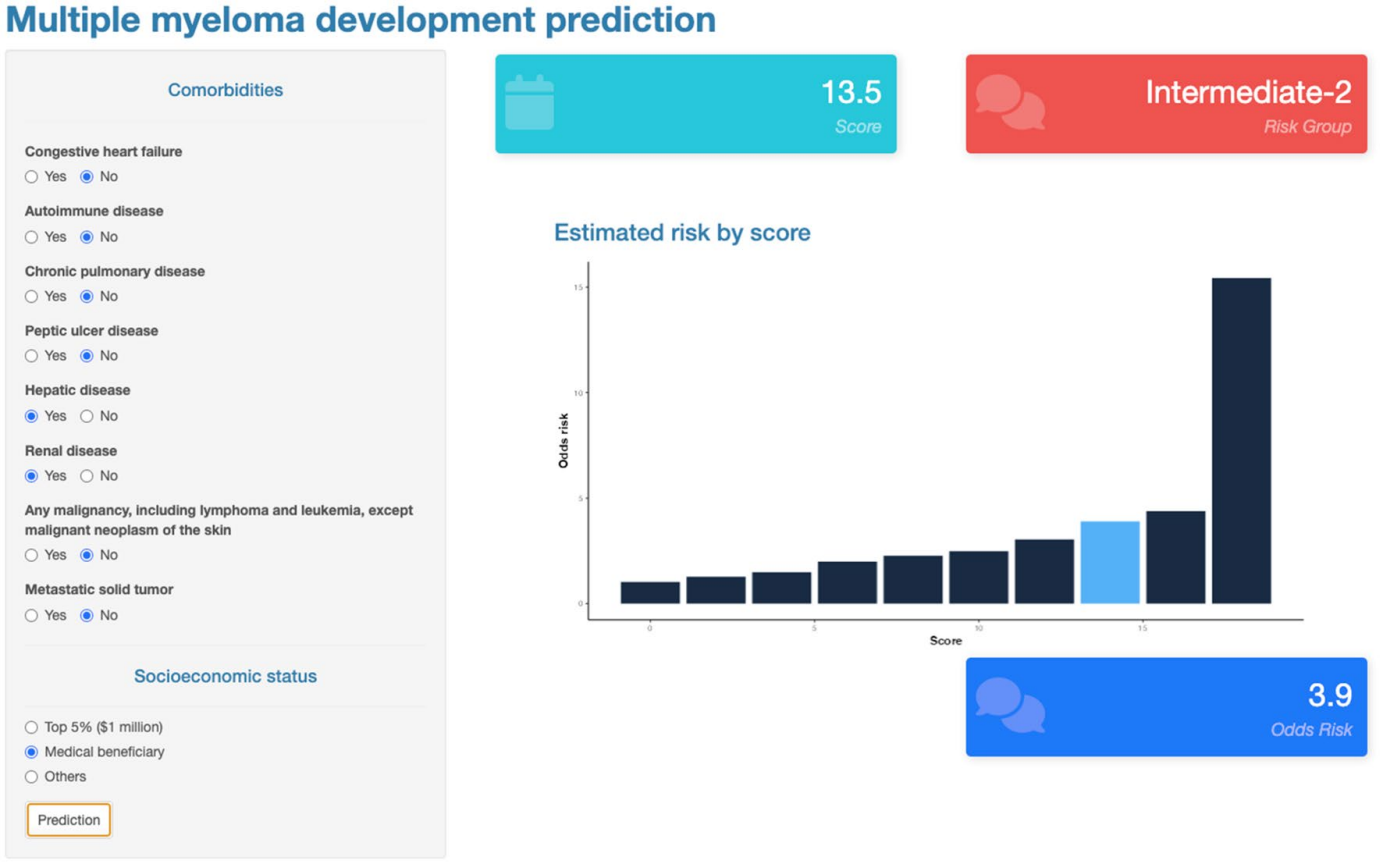
User interface of the open-source multiple myeloma risk prediction software.

The developed score model for MM risk prediction was implemented in R script and web-based R shiny application was developed to make it easy for clinician to use. A draft version of the software is now available at https://pipetapp.com/project/mm-risk/ with free unlimited access. Figure 3 shows example of the R shiny application interface to predict the risk of MM development.

## Discussion

In this study, epidemiological differences including sociodemographic characteristics and prior disease conditions were compared between MM patients and the matched general population who had not been diagnosed with MM. Further, a score model based on prior disease conditions and SES were developed to predict the risk of MM development and identify high-risk patients. The mean age at first diagnosis and proportions of male and females were comparable to previous results^18^. When compared with the age- and sex-matched control, MM patients showed significantly higher prevalence of 12 diseases and a medical beneficiary status before diagnosis. A multivariate logistic regression model identified 8 prior disease conditions and SES before diagnosis as significant predictors of MM development. Based on the result, the risk prediction score model was developed, and the total risk score was stratified into four groups for convenience: group without any risk (total risk score ≤ 0), 3 groups with 3 risk levels (intermediate-1, intermediate-2, and high-risk). These risk groups had ORs of 1.29, 3.07, and 4.62, respectively, when compared to the group without any risk. The ORs of a validation cohort, consisting of subjects who had with long-term observation period more than 5 years before diagnosis, showed similar result with that of original cohort, indicating that 1 year of collection period for prior disease conditions was sufficient for assessment. Finally, web-based application, which can be easily accessed online without a local installation, was developed to predict the risk to make it easier to use the model.

There have been a few reports investigating associations between prior disease conditions and the risk of developing MM, and previous reports that demonstrated relationships between some disease categories and incidence of MM support the results of the current study. T Choi et al.^19^ found an association between impaired renal function and risk of MM using health screening data in KNHIS database. The study found that patients with impaired renal function (GFR < 60 mL/min/1.73 m^2^) had 1.3-fold greater incidence of MM compared with those with normal function (GFR > 60 mL/min/1.73 m^2)^. In this study, renal disease had an overwhelmingly high OR of 2.06 (95% CI 1.9, 2.3) compared to other prior diseases which had ORs ranging from 1.03 to 1.18. Moreover, renal disease was found to have the shortest interval to MM diagnosis (40 months). It is well known that chronic renal disease contributes to increase of overall cancer risk, including MM^20,21^. Also, considering that increased gamma globulin production during early-stage MM can affect renal function, it is possible that renal dysfunction is a not a predisposing factor for MM development but pre-disease condition of MM, even though a 1 year of washout period was applied to prevent mistaking the pre-disease condition for the prior disease condition. However, including renal disease in the analysis was considered appropriate because the purpose of the study was to develop an assessment tool and to identify the high-risk population who need screening for MM. Thus, based on the current study result and the findings of T Choi et al., decreased renal function could be an important indicator of potential risk for MM development.

Also, several investigators have shown that a history of autoimmune disease is significantly associated with increased risks of MGUS and/or MM^22–24^. Ebba K. Lindqvist et al. found a significant association between a history of autoimmune disease and risk of MGUS/MM in a Swedish population-based cohort that included 19,112 MM patients, 5403 MGUS patients, and 96,617 population-based controls (OR of 2.1)^22^. In our study, even though MGUS patients were not included in this study, having autoimmune disease is significantly associated with increased MM risk showing OR of 1.2. For other prior disease conditions including congestive heart failure, hepatic disease, and other malignancies, this is the first report that showed MM risk quantitatively. The pathogenic mechanism linking these prior disease conditions to increased MM risk remains unclear; however, we believe that altered plasma cell dysfunction, germinal B cell hyperactivation after systemic inflammatory, and/or chronic antigenic stimulation with increased amyloid could be a common etiology driving the increased MM risk associated with these prior disease conditions^22^. Another plausible explanation is the potential identification of underestimation of systemic amyloidosis-driven organ failure as a comorbidity, which may have occurred over an extended period before the diagnosis of MM. Systemic amyloidosis is a rare disease entity, and it is estimated that 10–30% of cases are concomitant with MM^25,26^. Notably, it predominantly affects the heart (70%) and can subsequently involve the kidneys (60%) and liver (20%), in that order^27,28^. In other words, patients may have been exposed to systemic amyloidosis even before the MM diagnosis^29^, and the undiagnosed systemic amyloidosis could potentially account for various degrees of renal dysfunction and liver dysfunction within the case cohort.

Further research in this area may provide a deeper understanding of the pathogenesis of MM and lead to the development of new approaches for early detection and prevention of this disease.

In the study, individuals who had medical aid before the diagnosis showed significantly high OR (1.8) of MM development. In Korea, health insurance cost is determined based on the income and asset, and the medical aid beneficiary is provided to low-income families who are eligible through the National Basic Living Security Act, which accounts 2.8% of all Koreans. Thus, this result supports the previous report about association between low SES and high prevalence of MM because of the poor health status and environmental and occupational hazard in farmers and industrial workers, especially who had prolonged exposure to pesticides or other industrial chemicals^16,17^.

The importance of cancer screening cannot be emphasized enough in almost all types of non-hematologic malignancy in terms of the increases of both survival and cost-effectiveness by early cancer detection^30–32^. Both MM and MGUS, the latter of which is a premalignant condition of MM, can be detected by screening for abnormal protein in a 24-h urine sample using simple and non-invasive serum protein electrophoresis^10,33^. However, screening for detection of non-symptomatic MM (also referred to as smoldering MM) is not currently recommended, because the efficacy of early interventions have not been confirmed^33^. Nonetheless, as noted above, several studies have reported that early intervention with a combination of lenalidomide and dexamethasone provided benefits with respect to progression-free and overall survival in non-symptomatic MM patients at high risk of developing MM^34^. Based on this, it is increasingly clear that screening for hidden MM is necessary in selective populations at high risk of developing MM^35^. This study is the first nationwide cohort-based study to develop a predictive model of MM risk based on individual prior disease conditions and SES, identifying a high-risk group with a fivefold greater risk of MM development. The cost-effectiveness of this model should be assessed, and the study-defined high-risk group may be an optimal indication for future prospective studies.

There are several strengths of our study. First, although there have been a few epidemiologic studies addressing prior disease conditions and SES in MM patients, this is the first systematic nationwide study at a population level. Even though the potential for selection bias is inherent in the use of healthcare databases, we have taken steps to minimize its impact our result, by constructing the cohort based on nationwide health insurance data with a collection period of more than 10 years of all Korean citizens including both individuals with any history of medical claim and healthy individuals who had no history of medical claim. Our study control included subjects with varied health statuses rather than an exclusively healthy population, to include better reflect the general population and understand comorbities as risk factor for multiple myeloma. Also, prior disease conditions were categorized according to Charlson comorbidity index for standardization which has been well-known to clinicians and shown to have good clinimetric properties. Second, rigorous protocol was used to establish the cohorts including propensity score matching for demographics and the 1 year of washout period to prevent cases where MM-related symptoms may have been mistaken as prior disease conditions considering its median interval time from the first symptom to diagnosis^36^. Third, sub-cohort analyses were performed to show that the collection period in our study was relatively sufficient in terms of risk prediction. Finally, our study provides an easily applicable score model for predicting MM risk, which could help identify high-risk individuals who may benefit from the screening.

However, our study also has some limitations. First, as a retrospective study based on claims data, detailed clinical information such as disease severity or genetic factors which could affect the development of MM were not included. Also, because external validation was not performed, further studies are needed in different populations and settings, and caution should be taken when applying the findings to other populations.

## Conclusion

A scoring system based on prior disease conditions and SES was developed at a nationwide population level to predict the risk of developing MM. This scoring system could be a valuable tool to support clinicians in identifying potential candidates for MM screening.

## Materials and methods

### Data source & ethics

This study used data from the Korea National Health Insurance Service (KNHIS) database. The KNHIS is the medical public health insurance system that provides universal coverage to all citizens in South Korea (hereafter “Korea”). The KNHIS, which is comprehensive due to the mandatory national health insurance program, maintains a comprehensive set of databases containing health information for around 50 million Koreans including both healthy individuals and those with healthcare interactions, reflecting the general population's diversity. The databases include information regarding demographic variables such as age and sex, SES based on health insurance premium level, and healthcare data such as health screening results, medical claims, and mortality for the entire Korean population^37^. Thus, this database likely includes all medical data of almost all patients diagnosed with multiple myeloma in Korea and allows for identification of a control cohort for comparison. Because this study analyzed publicly available, anonymized, and de-identified data, the requirement for informed consent was waived. This study was approved and informed consent was waived by the Institutional Review Board of Seoul St. Mary’s Hospital, Seoul, Korea (No. KC21ZNSI0448) and was conducted in accordance with the Declaration of Helsinki and all other applicable regulations and guidelines.

### Study population and design

Flow diagram outlining selection of the case and control cohorts is shown in Fig. 1. We first included the patients who were diagnosed with multiple myeloma between January 2009 and December 2020 from the KNHIS database based on the International Classification of Diseases, Tenth Revision (ICD-10) using code C90 for multiple myeloma (Primitive case cohort). Then, the control cohort, who were not diagnosed with multiple myeloma and were matched to patients in case cohort based on birth date and sex, was selected from the KNHIS database (Primitive control cohort). The primitive case cohort was then processed to obtain the final case cohort according to the predefined protocol for the analysis: age over 18, presence of at least two times of MM main diagnosis. Also, patients who were diagnosed with multiple myeloma before 2011 was excluded to secure at least one year of data to collect the prior disease condition before the diagnosis of MM with one year of washing period. The final control cohort was established by matching the birth date- and sex- based propensity score with the final case cohort.

For patients in the case cohorts, the index date was defined as date of diagnosis of multiple myeloma. For individuals in the control cohorts, the index date of propensity-score-matched individuals in case cohorts was assigned as their index date. Thus, individuals who died before the index date in control cohort was also excluded for the analysis.

### Definitions

All baseline characteristics were collected from the data at the index date. Charlson comorbidity index (CCI), which has been conventionally and widely validated as a tool to predict the prognosis of patients with diverse medical illnesses, was employed to classify prior disease conditions of patients in a standardized manner. To collect the prior disease condition, diagnosis records (ICD-10 codes) of prior disease condition until 12 months before the index date were collected^38^. A washout period of 12 months was applied to exclude the possible MM-related symptoms or signs. Also, for each prior disease condition, reporting of the main diagnosis or at least two entries for sub-diagnoses was required, and the date of initial diagnosis was defined as that of prior disease condition diagnosis.

The SES was encoded by KNHIS as a numeric value based on the average monthly insurance premium, with 0 representing the medical aid group, and 1 to 20 representing evenly distributed percentiles (5% each). The initial groups were combined into three groups based on the analysis result: medical beneficiary, low-to-moderate (0–90% percentile), and high (top 10% percentile).

### Statistical analysis

For control cohort selection, a propensity score was determined using a logistic regression model, and birth date and sex were included as covariates with a caliper width equal to 0.25 of the standard deviation of the logit of the propensity score. The balance of propensity score was assessed by comparing the distribution of propensity score between the two groups and by calculating the standardized differences (less than 10%). To explore the difference in selected covariates between cohorts, baseline cohort characteristics were described and compared according to data type.

To evaluate the effect of the incidence of each prior disease condition on the risk of MM development, multivariate logistic regression was performed after stratifying the covariates according to its distribution and odds risk results. Variance Inflation Factor (VIF) was evaluated to address multicollinearity before multivariate logistic regression for each predictor included, and a VIF exceeding 5 were considered for removal or adjustment to ensure model stability and accuracy. To select the final model from the multivariate logistic regression, stepwise selection was performed, and Akaike information criteria (AIC) were used for the comparison. The model resulting from stepwise regression can include both significant and non-significant predictors since variables are chosen based on their effect on the AIC which does not depend solely on the corresponding *p*-value. Beta regression coefficients and odds ratios (ORs) with 95% confidence intervals (Cis) of each variable were calculated for the final model. Beta regression coefficients of variables were used for score development by assigning integer points for the prediction score. Individual risk estimates were based on the sum of the weighted scores for each variable.

The prediction scores were categorized into four groups considering the distribution and clinical efficiency, and ORs of each group were calculated. The ORs of subjects who had a collection period for prior disease condition longer than 5 years were also estimated and compared to previous result to ensure the validity of the score regarding the 1-year of collection period for prior disease condition. Considering the proportion and quality of missing data (less than 3%), complete case analysis was used for missing data. All statistical analyses were conducted using SAS version 9.4 (SAS Institute, Cary, NC, USA) and R version 4.0.3 statistical software (R Foundation for Statistical Computing, Vienna, Austria).

### Supplementary Information

Supplementary Tables.

## Data Availability

The data that support the findings of this study are available on request from the corresponding author. The data are not publicly available due to privacy or ethical restrictions.
