# Supplementary material for: Quantitative risk factor analysis of prior disease condition and socioeconomic status with the multiple myeloma development: nationwide cohort study
Source: Sci Rep. 2024 Feb 28;14:4885. doi: 10.1038/s41598-024-52720-1 (PMC10902317; doi:10.1038/s41598-024-52720-1)
Supplement: Supplementary file 1 — Supplementary Tables. [file 41598_2024_52720_MOESM1_ESM.docx]

**Supplementary Table S1. Time (month) from diagnosis of prior disease condition to diagnosis of multiple myeloma in the case cohort**

| **Prior disease condition** | **N** | **Interval between the first diagnosis (months)*** |
| --- | --- | --- |
| Congestive heart failure | 1282 | 53 [31, 84] |
| Peripheral vascular disease | 3439 | 62 [39, 93] |
| Cerebrovascular disease | 2548 | 63 [38, 93] |
| Autoimmune disease | 1124 | 63 [39, 89] |
| Chronic pulmonary disease | 7512 | 70 [44, 97] |
| Peptic ulcer disease | 6796 | 72 [46, 99] |
| Hepatic disease | 3986 | 72 [46, 98] |
| Renal disease | 622 | 40 [24, 71] |
| Diabetes without chronic complications | 4024 | 70 [43, 99] |
| Diabetes with chronic complications | 1896 | 69 [42, 99] |
| Any malignancy | 1604 | 62 [36, 92] |
| Metastatic solid tumor | 145 | 50 [24, 85] |

* All values were presented as median[Q1, Q3]

**Supplementary Table S2. Results of univariate logistic regression for potential risk factors of multiple myeloma**

| **Variables** | **Odds ratio (95% CI)** | **Regression**  **coefficient (95% CI)** | **P-value** |
| --- | --- | --- | --- |
| Myocardial infarction |  |  | 0.157 |
| No | Ref | Ref |  |
| Yes | 1.10 [0.96, 1.25] | 0.270 [0.22, 0.33] |  |
| Peripheral vascular disease |  |  | <0.001 |
| No | Ref | Ref |  |
| Yes | 1.08 [1.04, 1.13] | 0.077 [0.04, 0.12] |  |
| Congestive heart failure |  |  | <0.001 |
| No | Ref | Ref |  |
| Yes | 1.31 [1.24, 1.4] | 0.27 [0.22, 0.34] |  |
| Cerebrovascular disease |  |  | 0.028 |
| No | Ref | Ref |  |
| Yes | 1.05 [1.01, 1.1] | 0.049 [0.01, 0.1] |  |
| Dementia |  |  | 0.181 |
| No | Ref | Ref |  |
| Yes | 0.95 [0.88, 1.02] | -0.051 [-0.13, 0.02] |  |
| Hemiplegia or paraplegia |  |  | 0.236 |
| No | Ref | Ref |  |
| Yes | 0.91 [0.78, 1.06] | -0.094 [-0.25, 0.06] |  |
| Autoimmune disease |  |  | < 0.001 |
| No | Ref | Ref |  |
| Yes | 1.28 [1.19, 1.36] | 0.247 [0.17, 0.31] |  |
| Chronic pulmonary disease |  |  | < 0.001 |
| No | Ref | Ref |  |
| Yes | 1.23 [1.19, 1.27] | 0.207 [0.17, 0.24] |  |
| Peptic ulcer disease |  |  | < 0.001 |
| No | Ref | Ref |  |
| Yes | 1.12 [1.09, 1.16] | 0.113 [0.09, 0.15] |  |
| Hepatic disease |  |  | < 0.001 |
| No | Ref | Ref |  |
| Yes | 1.15 [1.11, 1.2] | 0.14 [0.1, 0.18] |  |
| Renal disease |  |  | < 0.001 |
| No | Ref | Ref |  |
| Yes | 2.26 [2.06, 2.47] | 0.815 [0.72, 0.9] |  |
| Diabetes without chronic complication |  |  | <0.001 |
|  | Ref | Ref |  |
|  | 1.09 [1.05, 1.13] | 0.086 [0.05, 0.12] |  |
| Diabetes with chronic complication |  |  | <0.001 |
|  | Ref | Ref |  |
|  | 1.16 [1.1, 1.22] | 0.148 [0.1, 0.2] |  |
| AIDS/HIV |  |  | 0.83 |
|  |  |  |  |
|  | 1.14 [0.27, 3.31] | 0.131 [-1.31, 1.2] |  |
| Any malignancy |  |  | < 0.001 |
| No | Ref | Ref |  |
| Yes | 1.24 [1.18, 1.31] | 0.215 [0.17, 0.27] |  |
| Metastatic solid tumor |  |  | < 0.001 |
| No | Ref | Ref |  |
| Yes | 1.4 [1.17, 1.66] | 0.336 [0.16, 0.51] |  |
| Socioeconomic status |  |  | < 0.001 |
| Medical beneficiary | 1.91 [1.76, 2.06] | 0.647 [0.57, 0.72] |  |
| Low-middle | Ref | Ref |  |
| High | 0.92 [0.89, 0.95] | -0.083 [-0.12, -0.05] |  |

**Supplementary Table S3. Variance inflation factor (VIF) of predictors used for multivariate analysis**

| **Predictors** | **VIF** | **Collinearity evaluation*** |
| --- | --- | --- |
| Congestive heart failure | 1.049 | Low |
| Peripheral vascular disease | 1.105 | Low |
| Cerebrovascular disease | 1.082 | Low |
| Autoimmune disease | 1.025 | Low |
| Chronic pulmonary disease | 1.100 | Low |
| Peptic ulcer disease | 1.113 | Low |
| Hepatic disease | 1.075 | Low |
| Renal disease | 1.030 | Low |
| Diabetes without chronic complications | 1.354 | Low |
| Diabetes with chronic complications | 1.328 | Low |
| Any malignancy | 1.095 | Low |
| Metastatic solid tumor | 1.074 | Low |

* Collinearity is considered low when VIF < 2
